# Lectin-Like Bacteriocins

**DOI:** 10.3389/fmicb.2018.02706

**Published:** 2018-11-12

**Authors:** Maarten G. K. Ghequire, Başak Öztürk, René De Mot

**Affiliations:** ^1^Centre of Microbial and Plant Genetics, KU Leuven, Leuven, Belgium; ^2^Leibniz-Institut DSMZ-Deutsche Sammlung von Mikroorganismen und Zellkulturen, Braunschweig, Germany

**Keywords:** LlpA, L-type pyocin, BAM complex, protein antibiotic, bacterial antagonism

## Abstract

Bacteria produce a diverse array of antagonistic compounds to restrict growth of microbial rivals. Contributing to this warfare are bacteriocins: secreted antibacterial peptides, proteins and multi-protein complexes. These compounds typically eliminate competitors closely related to the producer. Lectin-like bacteriocins (LlpAs) constitute a distinct class of such proteins, produced by *Pseudomonas* as well as some other proteobacterial genera. LlpAs share a common architecture consisting of two B-lectin domains, followed by a short carboxy-terminal extension. Two surface-exposed moieties on susceptible *Pseudomonas* cells are targeted by the respective lectin modules. The carboxy-terminal domain binds D-rhamnose residues present in the lipopolysaccharide layer, whereas the amino-terminal domain interacts with a polymorphic external loop of the outer-membrane protein insertase BamA, hence determining selectivity. The absence of a toxin-immunity module as found in modular bacteriocins and other polymorphic toxin systems, hints toward a novel mode of killing initiated at the cellular surface, not requiring bacteriocin import. Despite significant progress in understanding the function of LlpAs, outstanding questions include the secretion machinery recruited by lectin-like bacteriocins for their release, as well as a better understanding of the environmental signals initiating their expression.

## Introduction

Pseudomonads produce a diverse set of antagonism-mediating compounds that assist the elimination of rival microorganisms. A major subset of molecules contributing to this microbial fight are bacteriocins, ribosomally encoded antibacterial peptides and proteins that target bacteria closely related to the producing strain (Ghequire and De Mot, 2014). Bacteriocins assigned to different classes, based on molecular size and architecture, have been identified in a variety of *Pseudomonas* species (Lavermicocca et al., 2002; Parret et al., 2003, 2005; Barreteau et al., 2009; Fischer et al., 2012; Godino et al., 2015; Hockett et al., 2015). To date, research has primarily focused on *Pseudomonas aeruginosa* bacteriocins (termed pyocins) (Michel-Briand and Baysse, 2002). The bacteriocin armamentarium owned by pseudomonads varies from strain to strain (Ghequire and De Mot, 2014; Sharp et al., 2017). For different classes of bacteriocins, an evolutionary advantage has been demonstrated for bacteria secreting such compounds (Inglis et al., 2014; Ghoul et al., 2015; Godino et al., 2015; Dorosky et al., 2017; Príncipe et al., 2018).

Four main groups of *Pseudomonas* bacteriocins have been identified so far, all of which equally occur in other bacterial genera: tailocins, modular bacteriocins, B-type microcins and lectin-like bacteriocins (Supplementary Table S1). (i) Tailocins resemble contractile (R-type) or flexible (F-type) bacteriophage tails (Ghequire and De Mot, 2015; Scholl, 2017). Acquired from different phage sources, these high molecular-weight particles are synthesized from large gene clusters and are functional stand-alone units, lacking an accompanying phage head structure. (ii) Modular (S-type) bacteriocins represent a heterogeneous group of polymorphic toxins, and include a receptor-binding domain, a moiety assisting in membrane passage of target cells and a toxin domain (Ghequire and De Mot, 2014; Jamet and Nassif, 2015; Sharp et al., 2017). Self-inhibition due to toxin activity in bacteriocin producers is avoided by co-expression of dedicated immunity genes. These immunity partners form specific and high-affinity complexes with their cognate toxin domains, or reside in the cytoplasmic membrane to temporarily inhibit toxin activity during secretion (Rasouliha et al., 2013; Joshi et al., 2015; Ghequire et al., 2017b). To gain access to targeted pseudomonads, S-type bacteriocins take advantage of TonB-dependent outer-membrane proteins (White et al., 2017). (iii) B-type microcins are post-translationally modified peptides, interfering with DNA gyrase (Metelev et al., 2013). (iv) Lectin-like (further abbreviated as L-type) bacteriocins are composed of two monocot mannose-binding lectin domains and represent a fourth major class of *Pseudomonas* bacteriocins (Parret et al., 2003), with an unknown mode of action. In this review, we provide an overview summarizing current knowledge on the latter bacteriocin type, with emphasis on outstanding research questions.

## A Sugar-Binding Tandem Designed to Kill

Originally identified in banana rhizosphere isolate *Pseudomonas putida* BW11M1 (recently reclassified as *Pseudomonas mosselii* BW11M1) (Parret et al., 2003; Ghequire et al., 2016), the first lectin-like bacteriocin was termed LlpA (lectin-like putidacin A), and shown to possess selective genus-specific antagonistic activity, characteristic of bactericidal action. Later, LlpA bacteriocins were characterized in a number of other *Pseudomonas* species as well: *Pseudomonas protegens* (Parret et al., 2005), *Pseudomonas syringae* (Ghequire et al., 2012a) and *P. aeruginosa* (Ghequire et al., 2014; McCaughey et al., 2014) (Table 1). These bacteriocins are called “lectin-like” because all share an organization comprising two “monocot mannose-binding lectin” (MMBL) domains (B-lectin, Pfam PF01453), instead of a toxin-immunity module that is usually present in bacteriocins of similar size. The B-lectin domain is abundant in (monocot) plants (Van Damme et al., 1991; Pang et al., 2003; Gao et al., 2011; Pereira et al., 2015), but has also been found in fish (Tsutsui et al., 2003; de Santana Evangelista et al., 2009; Park et al., 2016; Arasu et al., 2017), fungi (Fouquaert et al., 2011; Shimokawa et al., 2012), slime molds (Barre et al., 1999) and sponges (Wiens et al., 2006). In those organisms a variety of antagonistic functions have been assigned to these lectins, including antifungal, antiviral and nematicidal activities (Ghequire et al., 2012b; Wu and Bao, 2013). A lectin-type bacteriocin with a distinct domain organization has been identified in a Gram-positive bacterium: albusin B of *Ruminococcus albus* 7 targets *Ruminococcus flavefaciens*, and consists of a B-lectin domain followed by a peptidase M15 domain (PF08291) (Chen et al., 2004). Genes encoding albusin B-like proteins have been retrieved in many other *R. albus* strains (Azevedo et al., 2015).

**Table 1:** Overview of functionally characterized proteobacterial LlpA bacteriocins.

Species	Strain	LlpA name	Size (AA)	Locus tag	Target spectrum	Reference
*Burkholderia cenocepacia*	AU1054	LlpA1_AU1054_ (or, LlpA_Bcen_1091_)	277^a^	Bcen_1091	Select bacteria belonging to *Burkholderia cepacia* complex, including several *B. ambifaria* strains	Ghequire et al., 2013a
	TAtl-371	LlpA88	277^a^	SAMN05443026_0088	Same target spectrum as LlpA1_AU1054_	Rojas-Rojas et al., 2018
*P. aeruginosa*	C1433	PyoL1	256	CDG56231	Select *P. aeruginosa* strains	Ghequire et al., 2014; McCaughey et al., 2014
*P. aeruginosa*	62	PyoL2	256	P997_04049	Select *P. aeruginosa* strains	Ghequire et al., 2014
*P. aeruginosa*	BWHPSA007	PyoL3	269^a^	Q020_03570	Select *P. aeruginosa* strains	Ghequire et al., 2014
*P. mosselii*	BW11M1	LlpA_BW11M1_ (or, LlpA_BW_)	276	AXZ07_RS10630	Mainly *P. syringae* (select strains), also some *P. fluorescens* and *P. putida* isolates	Parret et al., 2003; Ghequire et al., 2012a; McCaughey et al., 2014
*P. protegens*	Pf-5	LlpA1 (or, LlpA1_Pf-5_)	280	PFL_1229	Mainly isolates belonging to the *P. fluorescens* species group	Parret et al., 2005; Ghequire et al., 2012a
		LlpA2 (or, LlpA2_Pf-5_)	280	PFL_2127	Same target spectrum as LlpA1_Pf-5_	Parret et al., 2005
*P. syringae* pv. syringae	642	LlpA_Pss642_	290	COO_RS0109380	Select strains belonging to species groups of *P. aeruginosa*, *P. fluorescens*, *P. putida*, *P. syringae*	Ghequire et al., 2012a
*Xanthomonas citri* pv. malvacearum	LMG 761	LlpA_Xcm761_	248^a^	XAC0868	Select *Xanthomonas* strains	Ghequire et al., 2012a


*^a^size of the mature protein after cleavage of predicted Sec-dependent signal peptide.*

**FIGURE 1:**
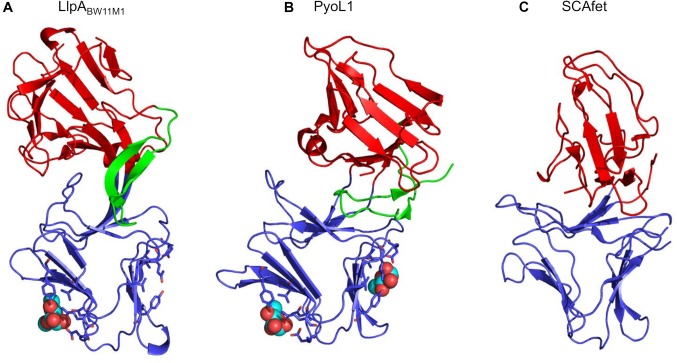
Ribbon diagram of structures from **(A)** LlpA from *P. mosselii* BW11M1 (LlpA_BW11M1_, PDB 3M7J), **(B)** LlpA from *P. aeruginosa* C1433 (PyoL1, PDB 4LED), **(C)** tandem MMBL plant protein SCAfet from *Scilla campanulata* (PDB 1DLP). The amino-terminal lectin domains are shown in red, the carboxy-terminal lectin domains in blue, and the carboxy-terminal extensions (absent in B-lectins from plants) in green. Side chains of coordinating residues constituting the D-rhamnose-binding lectin motifs in the carboxy-terminal lectin domains of LlpA_BW11M1_ and PyoL1 are shown as sticks. Methyl-mannose and D-rhamnose bound to LlpA_BW11M1_ and PyoL1, respectively, are shown as spheres.

The 3D structure of LlpA from strain BW11M1 (LlpA_BW11M1_) confirmed the architecture of two B-lectin domains as studied in monocot plants (Wright et al., 2000). Each module is stabilized by a central tryptophan triad. A short β-hairpin extension is present at the carboxy-terminus (Figure 1), but is absent from plant lectins (Ghequire et al., 2013b). The two domains form a rigid tandem due to β-strand swapping and intramolecular interactions (Figure 1). Such swapping can be equally noticed in dimers of single-domain MMBLs and tandem MMBLs from plants, though the relative orientation of each of the lectin domains in LlpAs may differ compared to (dimeric and tandem) MMBLs from plants (discussed in more detail in Ghequire et al., 2013b; McCaughey et al., 2014) (Figure 1). Plant B-lectin modules contain three carbohydrate-binding motifs with a consensus sequence QxDxNxVxY (Ghequire et al., 2012b; Wu and Bao, 2013), most of which are usually active, whereas these lectin motifs are often harder to discern in LlpAs (Ghequire et al., 2014). Motif conservation is mainly present in LlpA’s carboxy-terminal lectin domain. Sugar-binding properties are indeed linked to the latter domain, since intact QxDxNxVxY lectin motifs proved necessary to obtain a fully active bacteriocin. Nevertheless the affinity of LlpA_BW11M1_ for D-mannose and oligomannosides was observed to be quite low (Ghequire et al., 2013b), raising doubts about its biological significance. This issue was resolved after elucidating the structure of a lectin-like bacteriocin from cystic fibrosis isolate *P. aeruginosa* C1433, pyocin L1, that targets *P. aeruginosa* model strain PAO1 (McCaughey et al., 2014) (Figure 1). This L-type bacteriocin adopts a similar fold as LlpA_BW11M1_, but rather than binding D-mannose, pyocin L1 displays a much higher affinity for D-rhamnose, a 6-deoxy-D-mannose that is omnipresent in the common polysaccharide antigen (CPA) of *P. aeruginosa* (Lam et al., 2011). Two lectin motifs in the carboxy-terminal domain of pyocin L1 were ultimately shown to assist in this D-rhamnose binding, which equally appeared to be the case for LlpA_BW11M1_ (McCaughey et al., 2014). Following crystal soaks of LlpA_BW11M1_ with D-mannose, it was initially unclear whether one or two lectin motifs contributed to the protein’s sugar-binding properties (Ghequire et al., 2013b). More recently a third LlpA, LlpA1_Pf-5_ from *P. protegens* Pf-5, was equally found to depend on CPA for cellular killing (Ghequire et al., 2018b), suggesting that CPA likely is a common receptor among *Pseudomonas* LlpAs for target cell attachment. Carbohydrates present in O-antigen-specific lipopolysaccharide (LPS) are also used for cell surface docking by the tail fibers of R-type tailocins (Köhler et al., 2010; Kocincová and Lam, 2013; Ghequire et al., 2015; Buth et al., 2018), and some modular pyocins equally bind to CPA for target anchoring (McCaughey et al., 2016a). From a bacteriocin producer point of view, such targeting of LPS is a very attractive and effective strategy, since competitors become more susceptible to killing by detergents and permeable to antibiotics when attempting to escape from bacteriocin killing by LPS assembly loss (Ruiz et al., 2006; Falchi et al., 2018).

**FIGURE 2:**
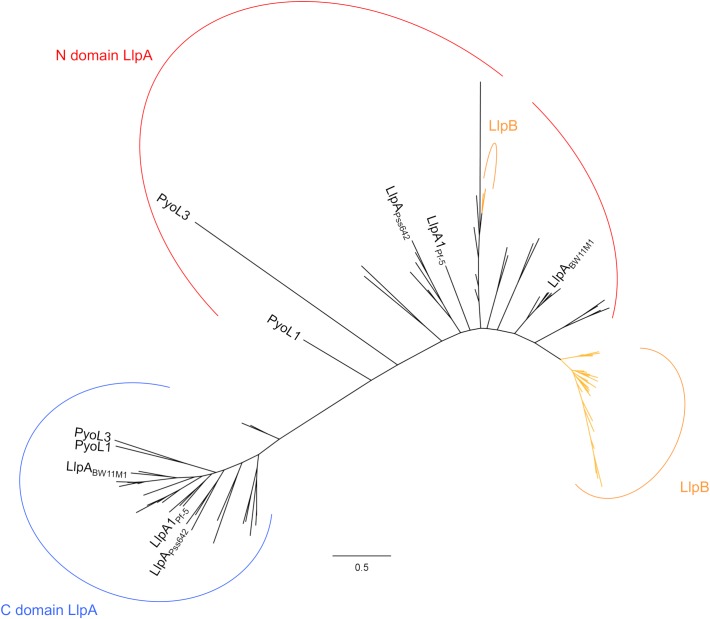
Maximum likelihood phylogenetic tree of individual B-lectin modules from *Pseudomonas* LlpAs and LlpBs. Amino-terminal domains (N) and carboxy-terminal domains (C) of LlpAs, and lectin domains of LlpBs are depicted by (a) red, blue and orange arc(s), respectively. Lectin domains originating from highly similar LlpA/LlpB sequences (>80% pairwise sequence id) are included as a single representative. Only characterized LlpAs are specified; species codes of other LlpAs and LlpBs, and bootstrap values (percentages of 1000 replicates) are not shown for clear distinction. A phylogenetic tree with complete annotations and bootstrap values is shown in the supplement (Supplementary Figure S1). Scale bar represents 0.5 substitutions per site.

Via activity/specificity assays using engineered LlpA chimers, it was further found that the amino-terminal lectin domain of LlpA accounts for target selection, regardless of the LPS-binding carboxy-terminal lectin module being present (Ghequire et al., 2013b). Phylogenetic analysis of individual lectin domains of characterized and putative LlpAs shows a clear clustering of each of the domains (Figure 2 and Supplementary Figure S1) (Ghequire et al., 2012b, 2014), which is in support of this domain-function dichotomy. Comparatively higher sequence conservation of the carboxy-terminal lectin domains [48% pairwise amino acid (AA) sequence identity (seq id)] results in a tighter clustering and advocates a more general function of the C-terminal lectin domain with regard to CPA binding (see above). Conversely, the amino-terminal domains have diverged more (∼39% pairwise AA seq id), and apparently evolved to hit different subsets of pseudomonads. Highly similar lectin-like pyocins PyoL1 and PyoL2 (86% pairwise AA seq id) display a divergent target spectrum, and differential bacteriocin residues primarily cluster at one patch of their amino-terminal lectin domains (Ghequire et al., 2014), which again supports the target-selective function of the amino-terminal lectin domain.

## Vandalizing an Essential Outer-Membrane Protein Assembly Machinery

Mutants of *P. aeruginosa* PAO1 defective in CPA biosynthesis do not become fully resistant to pyocin L1 killing (McCaughey et al., 2014). This suggests that a second receptor for L-type bacteriocin bactericidal action has to exist. Since CPA binding on its own cannot account for a cell death mechanism and given the lack of a distinct toxin domain, as present in modular bacteriocins, this was also expected. Recently, it was found that spontaneous mutants of *P. fluorescens* Pf0-1 become resistant to LlpA1_Pf-5_ killing when mutated in a surface-exposed loop of outer membrane protein (OMP) insertase BamA (Ghequire et al., 2018b). This essential protein consists of five polypeptide transport-associated (POTRA) domains and a carboxy-terminal β-barrel in the outer membrane, and interacts with lipoproteins BamB, BamC, BamD and BamE to constitute the BAM complex (Leyton et al., 2015; Noinaj et al., 2017). This machinery acts as the main catalyst for the insertion of new OMPs in the outer-membrane layer, with a key function attributed to BamA to partially unzip and create an open gate for a nascent OMP (Noinaj et al., 2014). The surface-exposed side of BamA is covered by three large loops (loops 4, 6, and 7) forming a dome-like structure (Ni et al., 2014). Mutations (single nucleotide polymorphisms, small in-frame deletions) in loop 6 (L6), allowed escape from LlpA1_Pf-5_ killing. In a similar experimental set-up using the reference strain PAO1, mutations in this same BamA loop were detected for spontaneous mutants resistant to PyoL1 killing (Ghequire et al., 2018b).

Depending on the *Pseudomonas* species group of interest, conservation in BamA may be moderate to very high. In *P. aeruginosa* in particular, BamA sequence conservation is nearly perfect, except for surface-exposed L6, the same loop in which mutations yielding bacteriocin resistance were detected. Furthermore, only a limited set of L6 sequence variants seem to exist in nature, that vary depending on the *Pseudomonas* species (group). Interestingly, a strong correlation between L6 sequence type and susceptibility to a certain LlpA was demonstrated for pyocins L1 and L2, and LlpA_BW11M1_ (Ghequire et al., 2018b). BamA indeed appears to be the key selectivity partner of LlpAs, since susceptibility to a particular LlpA is conferred upon an otherwise LlpA-resistant *Pseudomonas* by expression of a BamA with the corresponding LlpA-compatible L6 loop. LlpA producers escape from self-inhibition by expressing a BamA equipped with a different L6 than the one they are targeting (Ghequire et al., 2018b), an elegant mechanism preventing kin killing without the need for an immunity protein. Whereas modular bacteriocins typically display species-specific antagonism (Barreteau et al., 2009; Ghequire and De Mot, 2014; Godino et al., 2015; Ghequire et al., 2017a; Hockett et al., 2017), lectin-like bacteriocins (may) show genus-specific killing, which is explained by the occurrence of certain L6 sequence types in different *Pseudomonas* species (Ghequire et al., 2012a, 2018b). Taken together, the variation in surface-exposed loops of BamA proteins explains why different LlpAs target different subsets of pseudomonads. An interesting observation is that some effectors of contact-dependent growth inhibition (CDI) systems – polymorphic toxins released via a Type V secretion system mediating cell death by cell-to-cell contact (Willett et al., 2015; Chassaing and Cascales, 2018) – equally take advantage of BamA as a surface receptor (Aoki et al., 2008). As is the case for LlpAs, sequence polymorphism of BamA was found to determine susceptibility, although depending on two surface-exposed loops, L6 and L7 (Ruhe et al., 2013).

**FIGURE 3:**
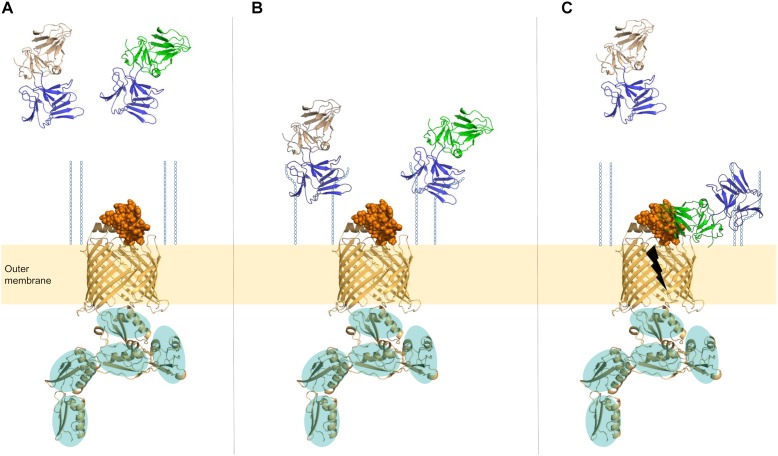
Proposed model of LlpA in interaction with its cell-surface receptors. **(A)** For BamA, the barrel in the outer membrane is shown in cartoon (orange), the surface-exposed loop L6 as orange spheres, and the five POTRA domains in the periplasm (cartoon, orange) are highlighted by teal ovals. The BamA sequence of *P. aeruginosa* PAO1 was used to construct the 3D-model (i-TASSER; https://zhanglab.ccmb.med.umich.edu/I-TASSER/). D-rhamnose units that are part of CPA are shown as blue hexagons (O-specific antigen of the lipopolysaccharide is not shown). CPA-binding domains of lectin-like bacteriocins (C-domains) are shown in blue, and amino-terminal lectin domains of LlpAs with different specificities are shown in wheat and green. **(B)** Lectin moieties in the carboxy-terminal domains of lectin-like bacteriocins attach to D-rhamnose residues present in CPA. **(C)** After/concurrent with CPA attachment, a stable bacteriocin-target interaction arises if L6 of BamA is recognized by LlpA. Killing action (black thunderbolt) is exerted in an unknown manner.

At this point, the molecular details of the interaction between LlpA and (L6 of) BamA remain elusive, and therefore it is currently unclear how exactly LlpA impairs the BAM function. It should be underlined that BamA is a dynamic protein given its role in OMP assembly: the integration of (a) new β-sheet(s) of a nascent OMP requires a destabilized and structurally rearranged seam of the BamA β-barrel (Doerner and Sousa, 2017). Conceivably, LlpA may impair the function of this lateral gate/exit pore located at the outer-membrane-periplasm interface by hindering structural reorganization, subsequently leading to a (lethal) downstream stress response (Figure 3). A role for the carboxy-terminal extension of LlpAs may also be anticipated since this stretch contains a number of hydrophobic residues that may mimic an elongated β-sheet (Figure 1) (Ghequire et al., 2013b; McCaughey et al., 2014). These residues may occupy BamA’s lateral pore, ultimately locking its function. LlpA’s rigid nature and the targeting of an essential protein strongly advocate a killing-upon-contact mechanism, in contrast to the killing-following-uptake of the more flexible S-type pyocins; the latter need partial unfolding to penetrate target cells via the β-barrel of TonB-dependent transporters and deliver their toxin load (White et al., 2017). A second unanswered issue concerns the observation that some (mainly fluorescent) pseudomonads are killed by different LlpAs (Ghequire et al., 2012a). Interestingly, a second *bamA* can be retrieved in the genomes of some pseudomonads, often (but not exclusively) belonging to the *P. fluorescens* group (Heinz and Lithgow, 2014). The physiological role of this BamA paralog remains undisclosed. The presence of a different L6 sequence type in this second BamA may explain the susceptibility of some pseudomonads to different LlpAs, though this is subject to experimental verification.

## LlpA: a Promising Protein Antibiotic for Bacteriocin Cocktails?

Their high potency, biodegradability and selective action makes bacteriocins a promising drug lead (Behrens et al., 2017; Ghequire and De Mot, 2018). Envisaging their therapeutic use, several mid-sized pyocins – including lectin-like bacteriocins – have been tested in a murine model of acute lung infection, and their high efficacy was demonstrated (McCaughey et al., 2016b). Interestingly, lectin-like pyocins are also amenable to large-scale production in plants (Paškevičius et al., 2017). The narrow spectrum of activity of bacteriocins nevertheless requires that several of these protein antibiotics are combined in a cocktail to guarantee coverage of the species diversity (Behrens et al., 2017). At this point it remains to be assessed (i) whether L-type pyocins could constitute a stable ingredient for such a cocktail, and (ii) whether the (low) mutation rate of L6 in BamA resulting in bacteriocin resistance (Ghequire et al., 2018b) is physiologically relevant, and whether this has any effect on bacterial fitness. Given the essential role of the BAM complex, several other therapeutic strategies are currently explored to interfere with its function, such as via monoclonal antibodies (Storek et al., 2018), peptides (Mori et al., 2012; Hagan et al., 2015) or peptidomimetic antibiotics (Urfer et al., 2016).

## Patchy Distribution of Lectin-Like Bacteriocin Genes

Lectin-like bacteriocin genes can be retrieved in genomes of virtually all *Pseudomonas* species, although sequence identity between LlpA homologs may be as low as 23% (pairwise AA identity among LlpAs), even if they originate from the same species (Supplementary Figure S2). The sole notable exception is *P. aeruginosa* in which L pyocins display high sequence similarity. Pyocins L1 and L2 share 86% amino acid sequence identity but exhibit a different target spectrum, coupled to the targeting of different BamA subsets (see above). Homology searches reveal the occurrence of a putative pyocin L4 in a small set of *P. aeruginosa* strains (e.g., in *P. aeruginosa* env100) that resembles pyocins L1 and L2 well (82 and 91% AA sequence identity, respectively). Possibly, pyocin L4 targets yet another L6 sequence type of BamA. On the contrary, pyocin L3 is much more diverged (∼26% sequence identity to L1/L2/L4). Overall, L pyocins can be retrieved in ∼4% of the assembled *P. aeruginosa* genomes, and a similar percentage (∼5%) of strains from other *Pseudomonas* species carry an *llpA* bacteriocin gene in their genome. LlpAs appear to occur more frequently in plant-associated and soil-dwelling pseudomonads, which may reflect a possible ancestral relationship with MMBL lectins from monocot plants. With few exceptions, *Pseudomonas* isolates only host one L-type bacteriocin gene in their genome, if any (Ghequire and De Mot, 2014). The latter observation strongly contrasts with modular S-type bacteriocins for which usually multiple representatives, albeit with different receptor-binding/toxin domain combinations, are present within a single *Pseudomonas* genome (Loper et al., 2012; Ghequire and De Mot, 2014; Sharp et al., 2017; Beaton et al., 2018). Bacteriocin sequences from strains encoding two LlpAs are usually dissimilar (30–52% pairwise AA id), arguing against a duplication event.

Genes encoding (putative) L-type bacteriocins have been recruited to a variety of loci, but are often present in tailocin and prophage clusters (Ghequire et al., 2015; Wang et al., 2016). As cargo genes, these *llpA* genes may spread via horizontal gene transfer. Lectin-like bacteriocin genes are hallmarked by a lower than host-average G + C content, a feature they share with modular bacteriocins (Mavrodi et al., 2009; Loper et al., 2012; Dingemans et al., 2016; Ghequire et al., 2017a,b; Ghequire and Öztürk, 2018). In general no clear correlation between LlpA phylogeny and *Pseudomonas* taxonomy can be made (except for *P. aeruginosa*, Supplementary Figure S2). Together with the observation that LlpA action may surpass species boundaries (Ghequire et al., 2012a), this further complicates the introduction of a thoughtful (re)classification of L-type bacteriocins. This lack of phylogeny-taxonomy correlation is reflected in the spectrum of BamA L6 variants that is not confined to species boundaries.

In addition to *Pseudomonas*, LlpA-like bacteriocins have also been described in another γ-proteobacterial genus, *Xanthomonas* (Ghequire et al., 2012a) and in the β-proteobacterium *Burkholderia* (Ghequire et al., 2013a; Rojas-Rojas et al., 2018) (Table 1), and putative L-type bacteriocins can also be detected in (select) genomes of a number of other genera, such as *Chromobacterium* and *Caballeronia* (both β-proteobacteria), though it remains unclear whether these proteins are bactericidal molecules as well. It also remains to be investigated whether LPS and BamA equally serve as receptors in susceptible strains of *Xanthomonas* and *Burkholderia*. D-rhamnose was previously detected as a constituent in lipopolysaccharides of *Xanthomonas* (Molinaro et al., 2003) and *Burkholderia* (Vinion-Dubiel and Goldberg, 2003; Karapetyan et al., 2006), although sugar-binding affinity for another oligosaccharide cannot be excluded a priori. Also in genomes of δ-proteobacteria (such as *Chondromyces*), Bacteroidetes (*Chryseobacterium*, *Spirosoma*, etc.) and diverse actinobacteria (*Arthrobacter*, *Pseudonocardia*, etc.), genes encoding a tandem MMBL protein can be retrieved. Furthermore, MMBL domains are often fused to one or more distinct domains, as highlighted earlier (Ghequire et al., 2012b). Overall, lectin-like bacteriocins seem confined to rather limited bacterial genera, thus representing a highly specialized tool, particularly apt for interbacterial warfare in plant-associated niches.

## LlpB: a Minimized Lectin-Like Bacteriocin?

In *Pseudomonas* genomes, a second type of B*-*lectin proteins can be discerned (Ghequire et al., 2012b) (Supplementary Table S1). These proteins, tentatively termed LlpBs, only host a single lectin domain, equally followed by a carboxy-terminal extension as is the case for LlpAs (size of ∼166 AA for LlpBs vs. ∼278 AA for LlpAs). The lectin domains of most of these LlpBs cluster on a distinct branch associated with the LlpA amino-terminal domain clade (Figure 2 and Supplementary Figure S1). Characteristic to B-lectins, these LlpBs consistently host a tryptophan triad and three potential sugar-binding motifs, one of which is well conserved and may be involved in carbohydrate binding. Antagonistic tests with select recombinant LlpB demonstrate that these proteins indeed display bactericidal action (Ghequire, 2013). Possibly the LPS-binding and target-selective function of LlpBs are condensed in a single lectin domain. One question arising is whether *llpA* genes evolved from *llpB* genes, or vice versa, or whether *llpA* and *llpB* genes were acquired independently. Overall, *llpB* genes can be retrieved in a variety of *Pseudomonas* species, but they appear to be absent from *P. aeruginosa* genomes. Similar to *llpA* genes, *llpB* genes are also present as cargos in tailocin or prophage clusters, for example in *Pseudomonas libanensis* DSM 17149 and *P. fluorescens* FF9, respectively, although this coupling appears to be much rarer.

## Secretion with a Sacrifice?

With the exception of a small subset of L-type pyocins (pyocin L3 and highly similar sequences) and *Burkholderia*/*Xanthomonas* representatives (Ghequire et al., 2014), LlpAs are not preceded by a Sec-dependent signal sequence motif to facilitate their secretion from producer cells. The same is true for S-type modular bacteriocins in *Pseudomonas* (Ghequire and De Mot, 2014), raising the question how these proteins can be released from producer cells? Group A colicin genes – encoding modular bacteriocins from *Escherichia coli* – are joined by a lysis module, typically located downstream of the bacteriocin gene, and these lipoprotein-encoding genes are co-expressed along with the colicins. For group B colicins, as is the case for *Pseudomonas* bacteriocins, a lysis gene is lacking (Cascales et al., 2007). However, for these colicins in particular it was found that the cellular release may be mediated by a prophage lysis module encoded elsewhere in the genome (Nedialkova et al., 2016; van Raay and Kerr, 2016). Conceivably, a similar strategy may be followed by LlpAs and other *Pseudomonas* bacteriocins as well, although this remains to be explored. The observation that L-type and other bacteriocin genes can often be retrieved within or in close proximity of tailocin and prophage gene clusters may facilitate co-expression of bacteriocin genes and lysis modules and co-inheritance, suggestive of such lysis “piggybacking” (Mavrodi et al., 2009; Loper et al., 2012; Ghequire et al., 2015, 2018a; Wang et al., 2016). Regardless of the mechanism used, the expression of lysis genes poses a burden on producer cells. For this reason, it is expected that only part of a *Pseudomonas* cell population secretes lectin-like bacteriocins, as demonstrated in *E. coli* for a colicin A/E2/E7 competition model (Bayramoglu et al., 2017).

## Stress-Triggered Retaliation?

The environmental cues controlling expression of L-type bacteriocins remain poorly understood to date. For LlpA from *P. mosselii* BW11M1 (Parret et al., 2003), and LlpA1_Pf-5_ and LlpA2_Pf-5_ from *P. protegens* Pf-5 (Parret et al., 2005), constitutive expression was observed, though it should be emphasized that bacteriocin-expression conditions may vary from strain to strain. Following exposure to UV light, LlpA_BW11M1_ expression is significantly enhanced. Similar observations have been made for several other (*Pseudomonas*) bacteriocins as well (Ghazaryan et al., 2014; Godino et al., 2015; Hockett and Baltrus, 2017; Turano et al., 2017), and such DNA-damaging conditions (e.g., via mitomycin C treatments) constitute a common strategy to induce bacteriocin overproduction. Screening of a *P. mosselii* BW11M1 transposon mutant library revealed that LlpA expression is reduced in a *recA* and *spoT* mutant background, confirming the UV-light-induced expression of LlpA (de los Santos et al., 2005). Following DNA-damaging treatment, RecA is activated leading to a stress response, which includes the activation of S-type pyocin expression in *P. aeruginosa* (Michel-Briand and Baysse, 2002; Ghequire and De Mot, 2014). Therefore, L-type bacteriocin expression may depend (in part) on a general regulatory mechanism triggering bacteriocin expression in pseudomonads. Similarly, colicins also depend on a SOS system to initiate their expression, mediated by LexA. This protein controls two overlapping SOS boxes, which prevents untimely colicin expression and cell lysis (Gillor et al., 2008; Žgur-Bertok, 2012; Fornelos et al., 2016).

**FIGURE 4:**
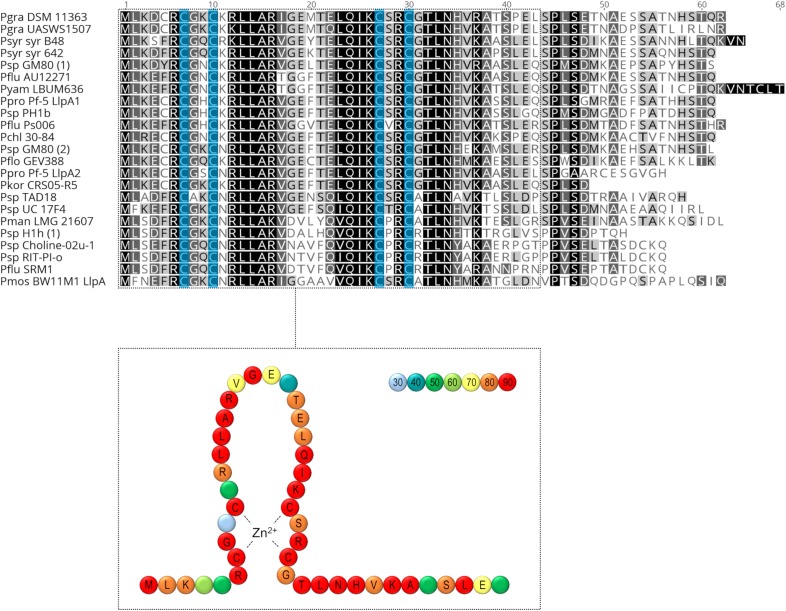
Multiple sequence alignment of *Pseudomonas* Com proteins (<90% pairwise AA sequence identity), encoded by genes preceding *llpA* genes. Differential shading reflects the sequence conservation. The conserved zinc-coordinating cysteine residues are highlighted in blue. In case two LlpAs are present in a single strain, preceding Com proteins (if present) are specified with (1) and (2). The zinc-finger domains (PF10122) of these Com proteins are marked by a dotted box and a schematic representation of the sequence conservation within this domain is shown under the alignment. Amino acids are depicted by beads and colored according to the (weighted) degree (%) of sequence conservation (legend) (AA sequences of all *llpA*-preceding Com proteins were included). In case sequence conservation is 70% or higher, the corresponding amino acid is identified inside the dot. The backbone of the scheme corresponds to the Com protein of the phage Mu. Pchl, *Pseudomonas chlororaphis*; Pflo, *Pseudomonas floridensis*; Pflu, *Pseudomonas fluorescens*; Pgra, *Pseudomonas graminis*; Pkor, *Pseudomonas koreensis*; Pman, *Pseudomonas mandelii*; Pmos, *Pseudomonas mosselii*; Ppro, *Pseudomonas protegens*; Psp, *Pseudomonas* sp.; Psyr syr, *Pseudomonas syringae* pv. syringae; Pyam, *Pseudomonas yamanorum*.

With the exception of *P. aeruginosa*, a large majority of L-type bacteriocin genes (∼90%) are preceded by a short gene encoding a putative zinc-finger-like protein (PF10122) (Ghequire et al., 2015), which is often not annotated in *Pseudomonas* genomes. Hallmarked by four conserved cysteine residues, this ∼60-AA protein is homologous to Com, a translational activator protein of bacteriophage Mu that initiates expression of the *mom* operon (Hattman et al., 1991; Witkowski et al., 1995). The amino-terminal region, which includes the coordinating cysteine residues, is largely conserved, whereas the Com tail may vary significantly in length (Figure 4). An interesting observation is that non-L-type bacteriocin genes present as tailocin cargos in pseudomonads may equally be preceded by such a *com-*like gene (Ghequire et al., 2015). Furthermore, in some strains that is also the case for the lytic enzyme that is part of the tailocin lysis cassette. *Com-*linked genes also appear in other *Pseudomonas* contexts, for instance, in *P. syringae* this gene is (often) linked to glycosyl hydrolase genes, including levansucrase homologs (Srivastava et al., 2012). The more general (regulatory) role of this Com protein remains to be explored.

## Concluding Remarks

Following the identification of the first lectin-like bacteriocin with a tandem lectin module architecture in a *P. mosselii* isolate, multiple LlpA representatives have been studied in a variety *Pseudomonas* species and other genera, largely facilitated by an ongoing release of genome sequencing data. The structure of LlpA, and the subsequent identification of CPA and BamA in particular as target receptors, are indicative of a novel killing mechanism that does not require protein import as observed for modular S-type bacteriocins. Undoubtedly a careful assessment of the interaction between LlpA and BamA will shed further light on the interference process exerted by these bacteriocins. Future research also needs to clarify the regulatory features for expression of this unusual group of bacteriocins, and elucidate how their secretion is accomplished. Given that the B-lectin module is present in proteins with different domain organizations, other lectin-like bacteriocin types likely exist.

## Author Contributions

MG, BÖ, and RDM wrote the manuscript. All authors revised and approved the manuscript.

## Conflict of Interest Statement

The authors declare that the research was conducted in the absence of any commercial or financial relationships that could be construed as a potential conflict of interest.
