# Supplementary material for: Lectin-Like Bacteriocins
Source: Front Microbiol. 2018 Nov 12;9:2706. doi: 10.3389/fmicb.2018.02706 (PMC6240691; doi:10.3389/fmicb.2018.02706)
Supplement: Supplementary file 1 [file Data_Sheet_1.docx]

Supplementary Material

Lectin-like bacteriocins

Maarten G. K. Ghequire^1^*, Başak Öztürk^2^, René De Mot^1^

^1^Centre of Microbial and Plant Genetics, KU Leuven, Kasteelpark Arenberg 20 bus 2460, 3001 Heverlee, Belgium

^2^Leibniz-Institut DSMZ-Deutsche Sammlung von Mikroorganismen und Zellkulturen, Braunschweig, Germany

*** Correspondence:** Corresponding Author: maarten.ghequire@ kuleuven.be

**Supplementary Table S1.** General overview of *Pseudomonas* bacteriocin classes with key characteristics.

| **Bacteriocin class** | **Subclass** | **Molecular weight (kDa)** | **Outer-membrane receptor** | **Self-immunity mechanism** | **Mechanism of action** |
| --- | --- | --- | --- | --- | --- |
| Tailocins | R-type (contractile) | 2.10^6^ – 10^7^ | Lipopolysaccharide | No | Membrane perforation |
|  | F-type (flexible) |  | ? |  | ? |
| Modular (S-type) bacteriocins | / | 32 – 90 | TonB-dependent transporters (FpvAI, FptA, HxuC, etc.) | Yes | Pore-formation, lipid-II degradation, or nuclease activity |
| B-type microcins | / | ~3.5 | ? | Yes | DNA gyrase inhibitor |
| Lectin-like bacteriocins | LlpA | ~30 | BamA, CPA | No | ? |
|  | LlpB | ~19 | ? |  | ? |

**Supplementary Figure S1.** Maximum likelihood phylogenetic tree of individual B-lectin modules from *Pseudomonas* LlpAs and LlpBs, depicted in Figure 2 with species-strain annotations. Amino-terminal domains and carboxy-terminal domains of LlpAs are marked with (N) and (C), respectively. MMBL domains of LlpBs are preceded by “B_” and highlighted in orange. Scale bar represents 0.6 substitutions per site, and bootstrap values (percentages of 1000 replicates) are shown at the branches. Species abbreviations: Paer, *Pseudomonas aeruginosa*; Pamy aes, *Pseudomonas amygdali* pv. aesculi; Pbra, *Pseudomonas brassicacearum*; Pcas, *Pseudomonas caspiana*; Pchl, *Pseudomonas chlororaphis*; Pcon, *Pseudomonas congelans*; Pflo, *Pseudomonas floridensis*; Pflu, *Pseudomonas fluorescens*; Pfre, *Pseudomonas frederiksbergensis*; Pgra, *Pseudomonas graminis*; Pmos, *Pseudomonas* mosselii; Pory, *Pseudomonas oryzihabitans*; Ppro, *Pseudomonas protegens*; Pput, *Pseudomonas putida*; Psp, *Pseudomonas* sp.; Psyr (syr), *Pseudomonas syringae* (pv. syringae); Pvir, *Pseudomonas viridiflava*.


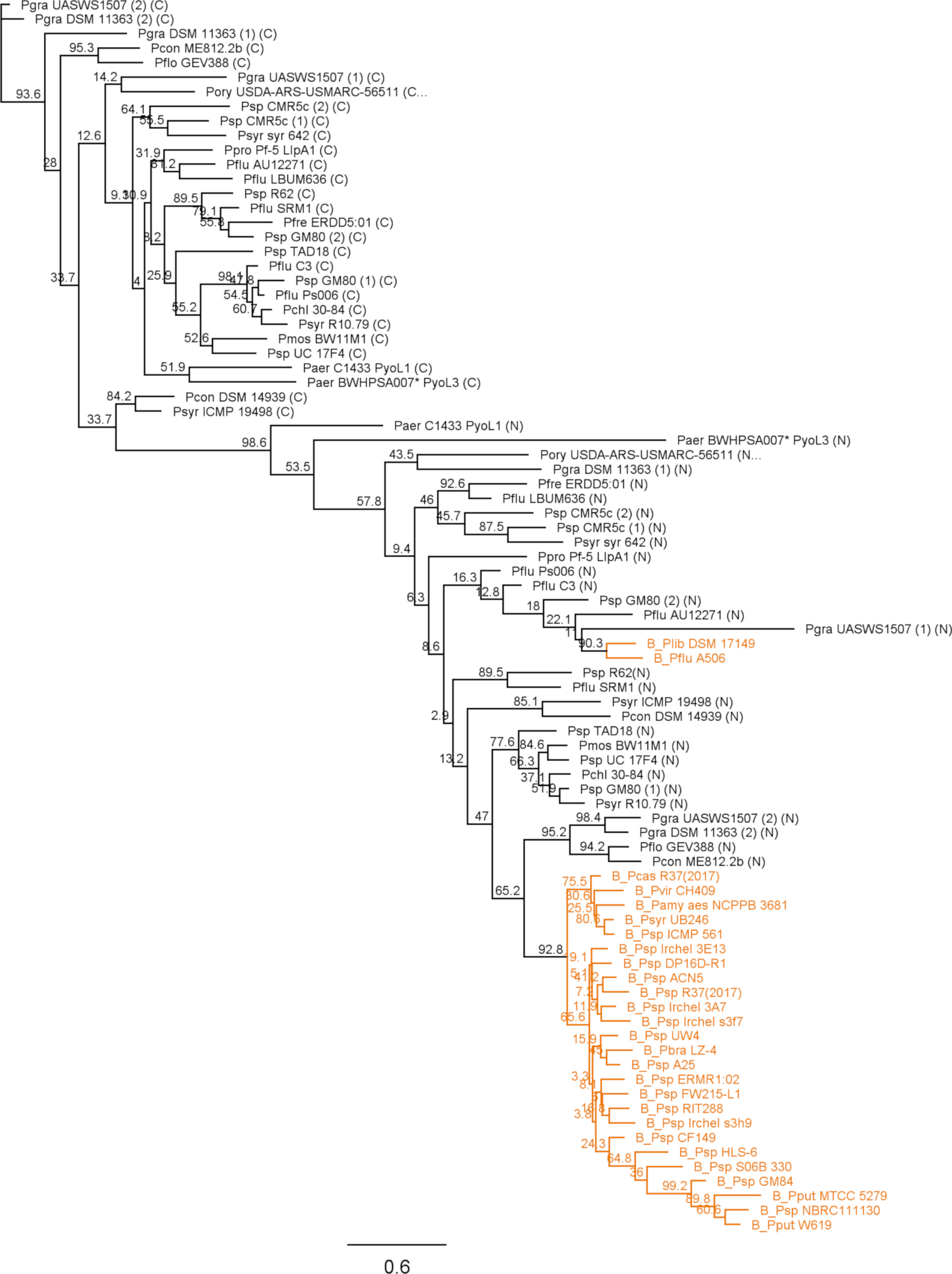


**Supplementary Figure S2.** Maximum likelihood phylogenetic tree of *Pseudomonas* LlpAs. Characterized LlpAs are blue. Highly similar sequences (>95% pairwise sequence id) are included as a single representative. In case two LlpAs are present in a single strain, bacteriocins are specified with (1) and (2). Scale bar represents 0.4 substitutions per site, and bootstrap values higher than 50 (percentages of 1000 replicates) are shown at the branches. The predicted signal peptide of pyocin L3 (marked with asterisk) was omitted. Species abbreviations: Paer, *Pseudomonas aeruginosa*; Pchl, *Pseudomonas chlororaphis*; Pcon, *Pseudomonas congelans*; Pflo, *Pseudomonas floridensis*; Pflu, *Pseudomonas fluorescens*; Pfre, *Pseudomonas frederiksbergensis*; Pgra, *Pseudomonas graminis*; Pman, *Pseudomonas mandelii*; Pmos, *Pseudomonas mosselii*; Pory, *Pseudomonas oryzihabitans*; Ppro, *Pseudomonas protegens*; Psp, *Pseudomonas* sp.; Psyr (dap/syr), *Pseudomonas syringae* (pv. daphniphylli/syringae).


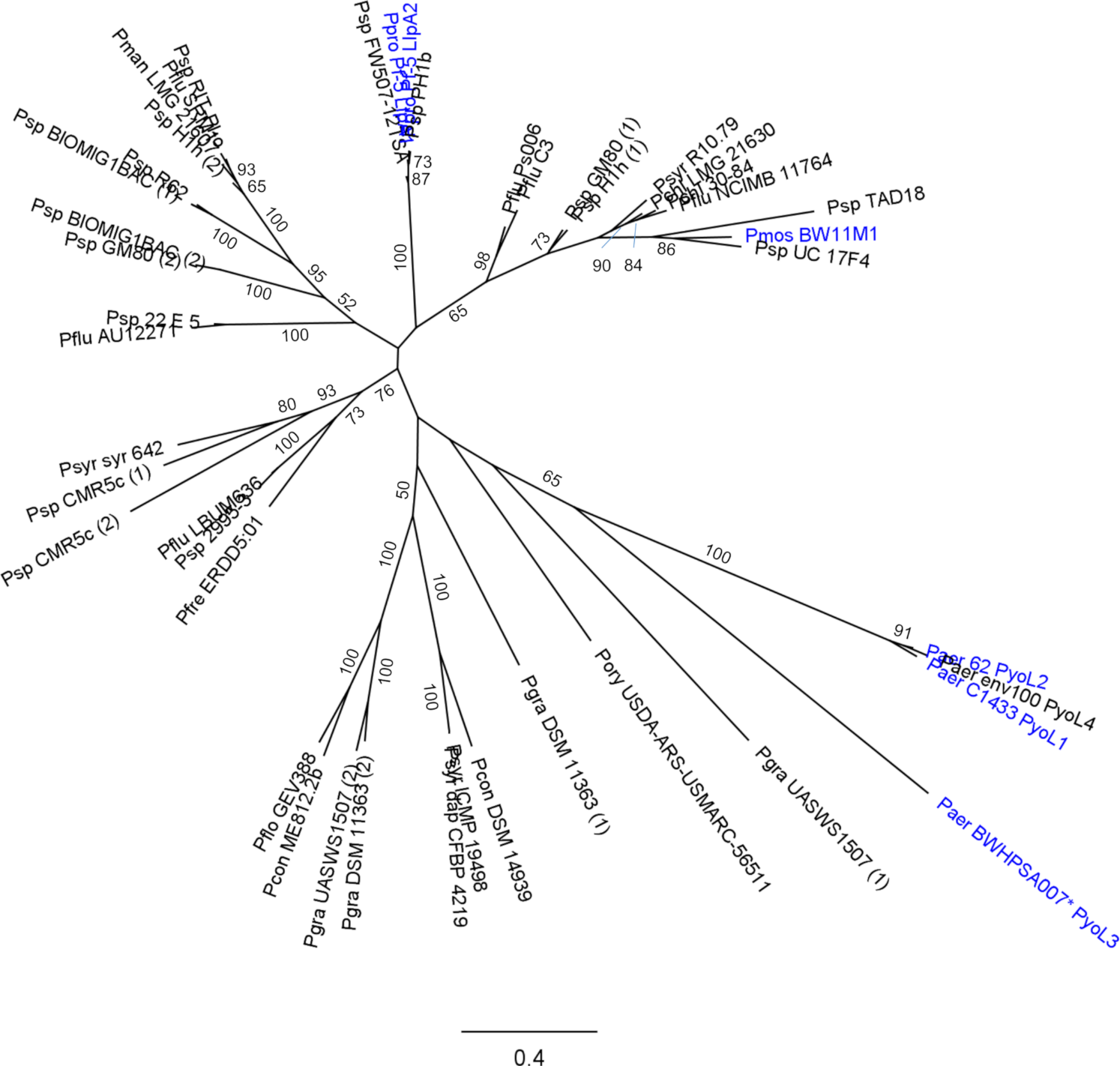


**
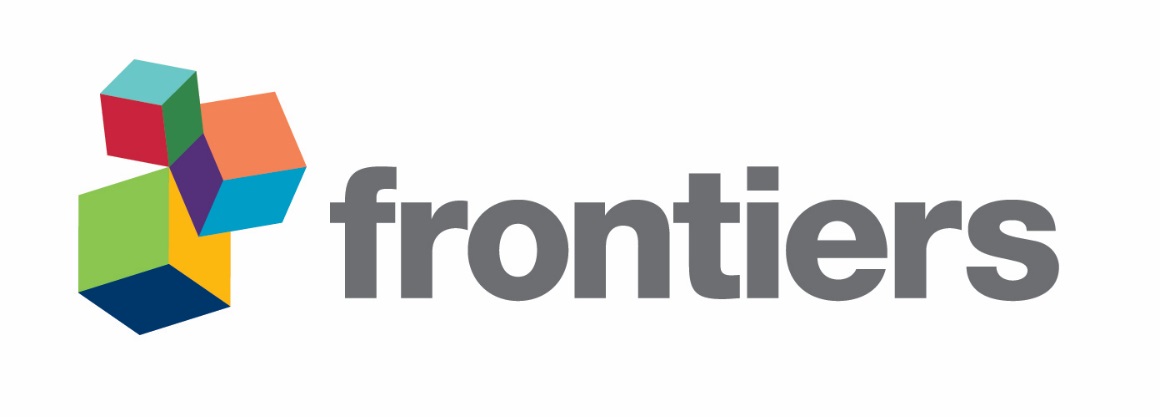
**
